# Supplementary material for: A novel interpretable machine learning system to generate clinical risk scores: An application for predicting early mortality or unplanned readmission in a retrospective cohort study
Source: PLOS Digit Health. 2022 Jun 13;1(6):e0000062. doi: 10.1371/journal.pdig.0000062 (PMC9931273; doi:10.1371/journal.pdig.0000062)
Supplement: S1 Text — (PDF) [file pdig.0000062.s001.pdf]

Supplementary table and figures

Table A. The LACE index.

**Variable**	**Interval**	**Point**
Inpatient length of stay ("L")	<1	0
	1	1
	2	2
	3	3
	4-6	4
	7-13	5
	≥14	7
Acute (emergent) admission ("A")	Yes	3
Charlson comorbidity index ("C")	0	0
	1	1
	2	2
	3	3
	≥4	5
Visits to emergency department during previous 6 months ("E")	0	0
	1	1
	2	2
	3	3
	≥4	4

Fig A. Parsimony plot on the validation set using ShapleyVIC variable ranking, based on all 41 variables.

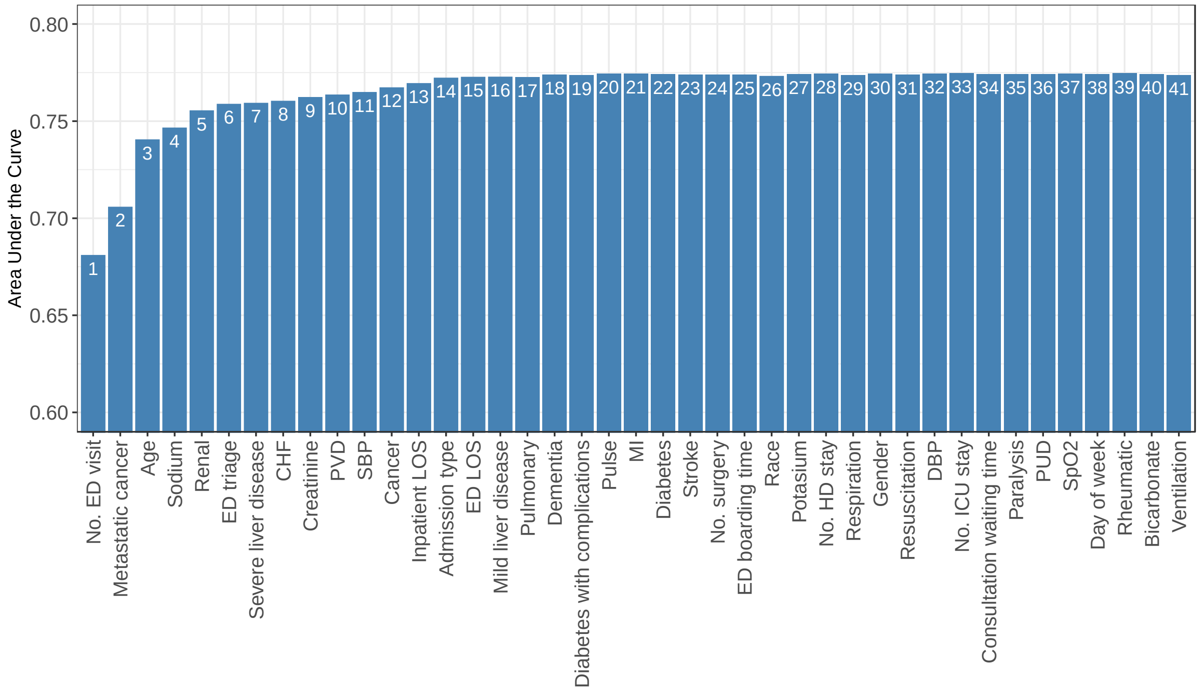

Ventilation was consistently estimated with zero ShapleyVIC value in all 350 models analyzed, as too few cases had ventilation and therefore this variable made little contribution to accurate prediction of the outcome. Hence, ventilation had non-significant overall importance and was assigned the lowest rank among all 41 variables. Number of ED visit is within 6 months before current inpatient stay. Number of surgery, ICU stay and HD stay are within 1 year before current inpatient stay.

CHF: Congestive heart failure; DBP: diastolic blood pressure; ED: emergency department; HD: high dependency ward; ICU: intensive care unit; LOS: length of stay; MI: Myocardial infarction; PVD: Peripheral vascular disease; PUD: Peptic ulcer disease; SBP: systolic blood pressure; SpO_2: blood oxygen saturation.

Fig B. Parsimony plot on the validation set using XGBoost-based variable ranking. ED triage was ranked 3rd but contributed little to model performance, whereas the number of ED visits and metastatic cancer have been found to be important contributors in the main analyses but were only ranked 8th and 40th, respectively.

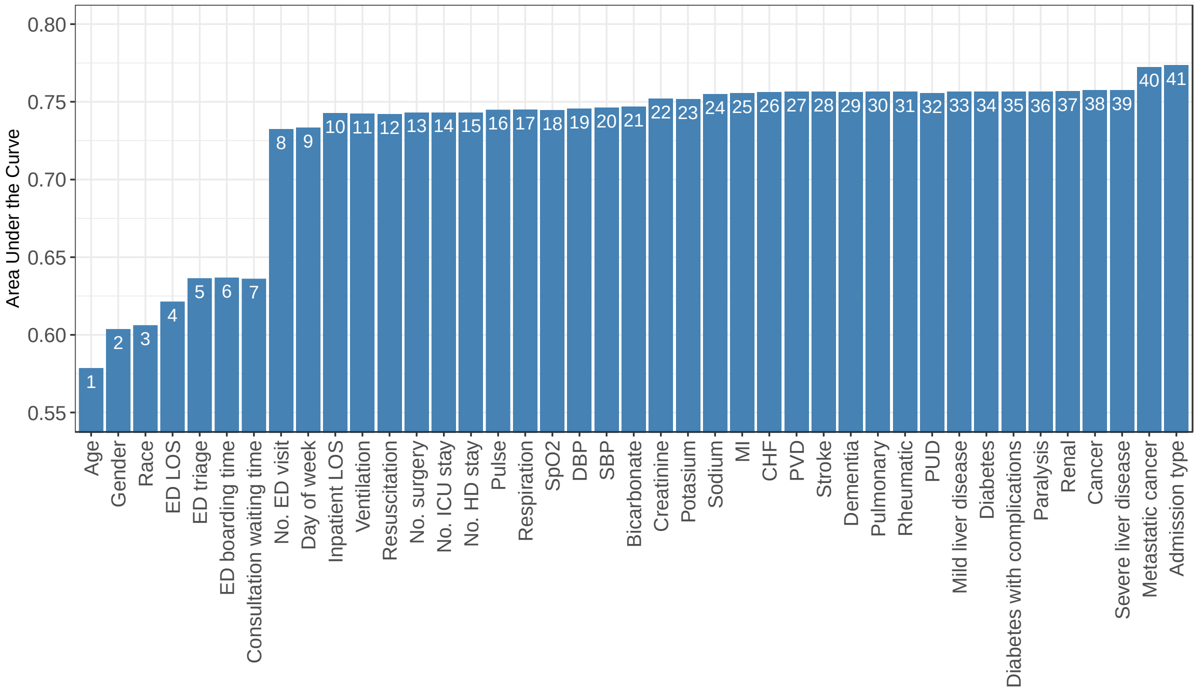

Number of ED visit is within 6 months before current inpatient stay. Number of surgery, ICU stay and HD stay are within 1 year before current inpatient stay.
CHF: Congestive heart failure; DBP: diastolic blood pressure; ED: emergency department; HD: high dependency ward; ICU: intensive care unit; LOS: length of stay; MI: Myocardial infarction; PVD: Peripheral vascular disease; PUD: Peptic ulcer disease; SBP: systolic blood pressure; SpO_2: blood oxygen saturation.
